# The prognostic relevance of urokinase-type plasminogen activator (uPA) in the blood of patients with metastatic breast cancer

**DOI:** 10.1038/s41598-018-37259-2

**Published:** 2019-02-19

**Authors:** Malgorzata Banys-Paluchowski, Isabell Witzel, Bahriye Aktas, Peter A. Fasching, Andreas Hartkopf, Wolfgang Janni, Sabine Kasimir-Bauer, Klaus Pantel, Gerhard Schön, Brigitte Rack, Sabine Riethdorf, Erich-Franz Solomayer, Tanja Fehm, Volkmar Müller

**Affiliations:** 1 Department of Gynecology and Obstetrics, Asklepios-Klinik Barmbek, Hamburg, Germany; 2 Department of Gynecology, University Medical Center Hamburg-Eppendorf, Hamburg, Germany; 3 Department of Obstetrics and Gynecology, University Hospital Leipzig, Leipzig, Germany; 4 Department of Gynecology and Obstetrics, University Hospital Erlangen, Comprehensive Cancer Center Erlangen-EMN, Friedrich-Alexander University Erlangen-Nuremberg, Erlangen, Germany; 5 Department of Obstetrics and Gynecology, University Hospital Tübingen, University of Tübingen, Tübingen, Germany; 6 Department of Gynecology and Obstetrics, University Hospital Ulm, Ulm, Germany; 7 Department of Obstetrics and Gynecology, University Hospital Essen, University of Duisburg-Essen, Essen, Germany; 8 Department of Tumour Biology, University Medical Center Hamburg-Eppendorf, Hamburg, Germany; 9 Department of Medical Biometry and Epidemiology, University Medical Center Hamburg-Eppendorf, Hamburg, Germany; 10 Department of Gynecology and Obstetrics, Saarland University Hospital, Homburg/Saar, Germany; 11 Department of Obstetrics and Gynecology, Heinrich-Heine-University Düsseldorf, Düsseldorf, Germany

## Abstract

In breast cancer (BC), elevated levels of urokinase-type plasminogen activator (uPA) in tumor tissue have been confirmed as a strong prognostic factor in level-of-evidence-1 studies. The aim of the present study was to evaluate the clinical relevance of uPA levels in serum of metastatic BC patients and to compare uPA with other blood-based biomarkers. 252 patients were enrolled in this prospective, multicentre study. Blood samples were collected before begin of first-line or later-line systemic treatment. Serum uPA was quantified by a commercially available ELISA. Circulating tumor cells (CTCs) were detected using CellSearch; other biomarkers (EGFR, VEGF, HER2, RAS p21, TIMP1, CAIX) by ELISA. Using the ROC analysis, the optimal cut-off value (determined by the Youden index) of serum uPA was 2.52 ng/ml. Using this value, 26% of patients had elevated uPA levels. Patients with visceral metastasis and more than one metastatic site were significantly more likely to present with elevated uPA levels. CTC status, serum HER2, RAS p21, CAIX, TIMP1 and VEGF correlated significantly with uPA levels. Elevated uPA levels predicted shorter overall and progression-free survival in univariate analysis (median OS: 7.5 months [95%-CI 4.5–10.5 months] vs. not reached, p < 0.001; PFS: 4.8 [95%-CI: 3.1–6.5] vs. 9.1 [7.4–10.8] months, p < 0.001). In multivariate analysis, elevated uPA, presence of ≥5 CTCs, elevated RAS p21, higher grading and higher line of therapy were independent predictors of shorter OS, while elevated CTC counts, higher line of therapy and negative estrogen receptor status were independent predictors of shorter PFS. In conclusion, elevated uPA levels independently predict reduced overall survival and improved prognostication in patients with known CTC status. Whether high serum uPA might identify patients most likely to benefit from therapies targeting uPA, remains to be evaluated in future trials.

## Introduction

Metastatic spread involves several crucial processes, including detachment of cancer cells from their original site, migration and invasion into the surrounding tissue^1^. This last step requires the release of proteolytic enzymes which facilitate breakdown of extracellular matrix (ECM) and basement membranes^2^. In this context, the role of plasminogen activators (PA) has been extensively studied. Two main natural activators, the tissue-type (tPA) and the urokinase-type (uPA), both catalyze the conversion from plasminogen to plasmin; the first one is assumed to play a major role in thrombolysis while the latter one generates plasmin in events involving cancer metastasis^2^. Plasmin, as a strong proteolytic enzyme, is able to degrade or remodel proteins building the ECM, such as fibrin, fibronectin, laminin and vitronectin and thus create a localized microenvironment of matrix degradation, facilitating migration and invasion of cancer cells. To restrain its proteolytic activity, uPA can be controlled by a negative feedback loop, mediated by plasminogen activator inhibitors 1 and 2 (PAI-1 and PAI-2).

In breast cancer (BC), uPA and PAI-1 can be measured in tumor tissue using various methodologies, including ELISA and immunohistochemistry at the protein level and RT-PCR at mRNA level. Numerous trials reported that elevated uPA and PAI-1 levels predict poor clinical outcome. Since then, the combined detection of uPA/PAI-1 have been confirmed as a strong prognostic factor in level-of-evidence-1 studies in node-negative BC patients^3,4^ and the biomarker has been incorporated into national and international guidelines^5,6^. Despite high quality of evidence, the recent update of the American Society of Clinical Oncology (ASCO) Clinical Practice Guideline on biomarkers allows the use of uPA/PAI-1 to guide decisions on adjuvant systemic therapy in hormone receptor-positive HER2-negative patients but the strength of recommendation was qualified as weak^5^ and the use is limited due to the requirement for fresh-frozen tissue and the introduction of standardized gene signatures^7,8^. As uPA can be shed from tumor cells into the blood stream, the potential use of circulating uPA in plasma or serum has been first explored in the late 1980’s and early 1990’s, when several groups have found higher uPA levels in patients with malignant diseases than in healthy controls^9,10^. Limited data are so far available on the prognostic relevance of circulating uPA in cancer patients, with conflicting results reported in various entities^11–16^.

The aim of the present study was to evaluate the clinical relevance of uPA levels in serum of metastatic BC patients and to compare uPA with other blood-based biomarkers, most importantly the circulating tumor cells (CTCs) that were determined prospectively in a large multicentre cohort.

## Results

### Patients’ characteristics

Clinical-pathological data of 252 metastatic BC patients enrolled in the study are summarized in Table 1. Blood sample was collected at time of first diagnosis of metastatic disease in 39% of patients, in the remaining 61% of cases at time of cancer progression. The majority of patients had visceral metastasis (86%). In 49.8% of patients at least five CTCs per 7.5 ml of peripheral blood were detected.

**Table 1:** Distribution of the study patients according to serum uPA in correlation to clinical-pathological characteristics and other blood-based biomarkers (significant values are shown in bold).

	Total	uPA ≥2.52 ng/ml n (%)	p-value
**Overall**	252	65 (26%)	
**Menopausal status**			0.22
Premenopausal	69	14 (20%)	
Postmenopausal	183	51 (28%)	
**Metastatic site**			**0.036**
Visceral +/− bone	217	61 (28%)	
Bone only	35	4 (11%)	
**Extent of metastatic disease**			**0.016**
One site	85	14 (17%)	
Multiple sites	167	51 (31%)	
**Therapeutic setting**			0.075
1st-line	98	19 (19%)	
2nd-line or higher	153	45 (29%)	
**Grading**			0.607
G1-G2	135	34 (25%)	
G3	103	29 (28%)	
**Tumor subtype**			0.063
Triple-negative	37	13 (35%)	
HR-pos HER2-neg	106	19 (18%)	
HER2-positive	76	22 (29%)	
**ER status**			0.467
Negative	76	22 (29%)	
Positive	175	43 (25%)	
**PR status**			0.293
Negative	102	30 (29%)	
Positive	149	35 (24%)	
**HER2 status**			0.283
Negative	143	32 (22%)	
Positive	76	22 (29%)	
**Circulating tumor cells**			**0.008**
<5 CTCs/7.5 ml	122	22 (18%)	
≥5 CTCs/7.5 ml	122	40 (33%)	
**Serum HER2**			**0.001**
<15 ng/ml	131	22 (17%)	
≥15 ng/ml	119	42 (35%)	
**Serum RAS p21**			**0.003**
<452 pg/ml	222	50 (23%)	
≥452 pg/ml	29	14 (48%)	
**Serum CAIX**			**<0.001**
<506 ng/ml	162	28 (17%)	
≥506 ng/ml	90	37 (41%)	
**Serum TIMP1**			**<0.001**
<454 ng/ml	183	29 (16%)	
≥454 ng/ml	69	36 (52%)	
**Serum VEGF**			**0.001**
<367 pg/ml	189	39 (21%)	
≥367 pg/ml	63	26 (41%)	
**Serum EGFR**			0.678
<73 ng/ml	189	50 (27%)	
≥73 ng/ml	63	15 (24%)	

Abbreviations: HR – hormone receptor.

### Serum uPA detection and determination of the cut-off

**Figure 1:**
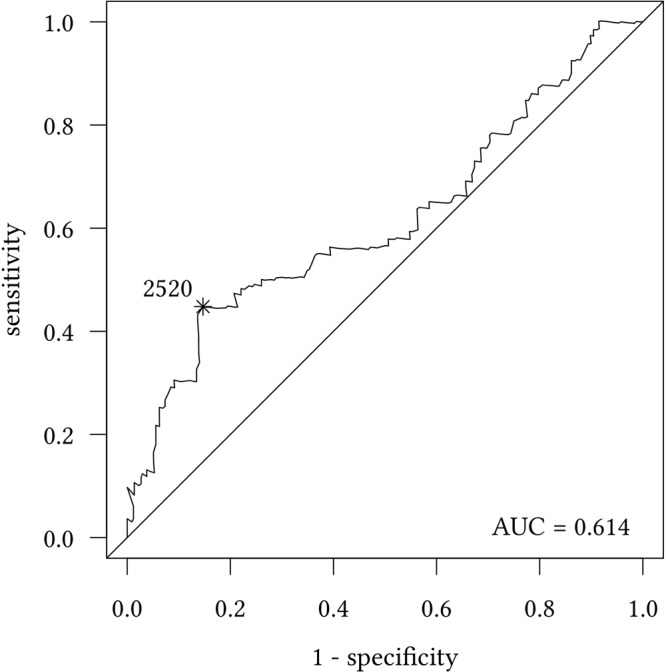
Survival ROC for serum uPA and 1-year-survival (Youden-Index for the cut-off value of 2.52 ng/ml: sensitivity 44.8%, specificity 85.3%).

Levels of serum uPA were measured in 252 patients, with a median concentration of 1.92 ng/ml (mean 2.15 ng/ml, range 0.21–16.06 ng/ml, standard deviation 1.37 ng/ml). Since no established cut-off value for serum uPA was established so far, we performed a ROC analysis for the 1-year-survival to evaluate possible cut-off values (Fig. 1). The optimal cut-off using the Youden Index was 2.52 ng/ml. Using this value, 26% of the patients had elevated uPA levels.

No correlation was found between uPA levels and tumor subtype or grading (Table 1). Patients with visceral metastasis and more than one metastatic site were significantly more likely to present with elevated uPA. CTC status correlated significantly with uPA levels (p = 0.008). With regard to other blood-based biomarkers, a significant association was observed between uPA and serum HER2 (p = 0.001), RAS p21 (p = 0.003), CAIX (p < 0.001), TIMP1 (p < 0.001) and VEGF (p = 0.001).

### Univariate survival analysis

**Figure 2:**
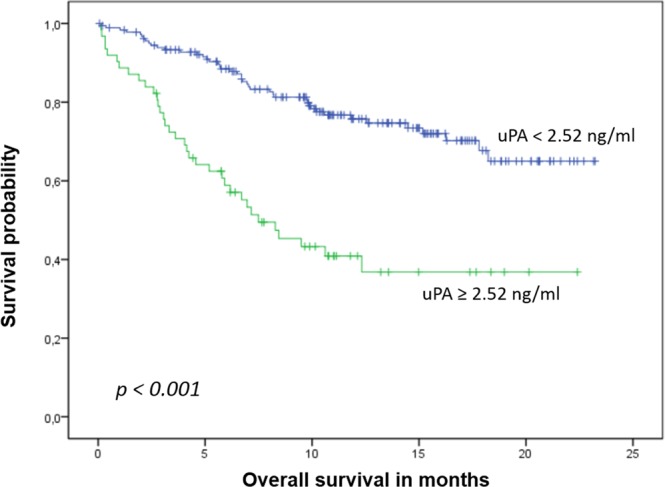
Kaplan-Meier plot of overall survival according to uPA levels.

**Figure 3:**
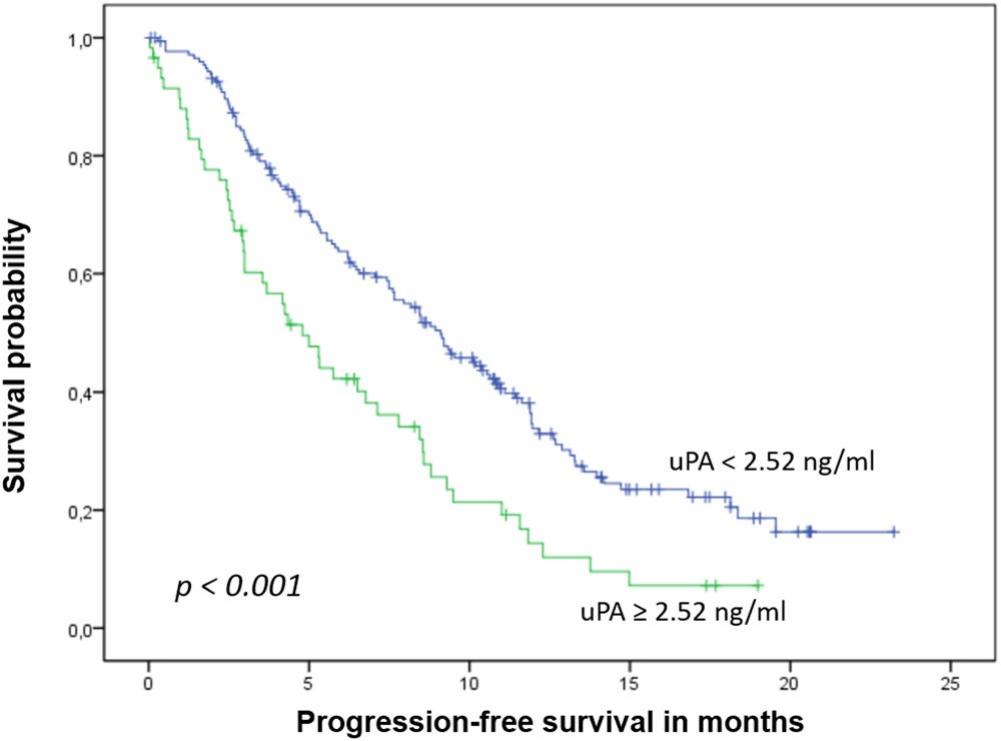
Kaplan-Meier plot of progression-free survival according to uPA levels.

**Figure 4:**
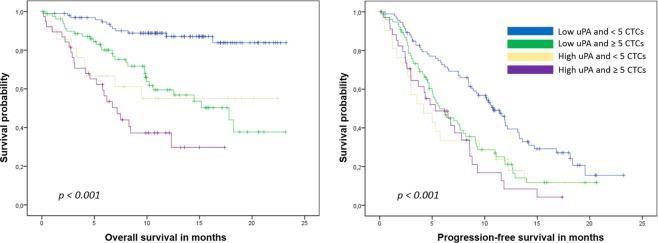
Kaplan-Meier plot of overall and progression-free survival according to circulating tumor cells and uPA levels.

During a median follow up of 19 months, 85 patients died and 183 were diagnosed with progressive disease. Patients with elevated uPA levels had significantly shorter overall and progression-free survival than patients with non-elevated levels (median OS: 7.5 months [95%-CI 4.5–10.5 months] vs. not reached, p < 0.001, Fig. 2; PFS: 4.8 [95%-CI: 3.1–6.5] vs. 9.1 [7.4–10.8] months, p < 0.001, Fig. 3). No significant impact on survival was observed when median or the lower quartile were considered as cut-off values (OS: p = 0.155 for the 25^th^ percentile and p = 0.146 for the median; PFS: p = 0.075 for the 25^th^ percentile and p = 0.248 for the median). Patients with elevated uPA and ≥5 CTCs had the shortest OS, while patients with non-elevated uPA and <5 CTCs had the longest OS (Table 2, Fig. 4). In the subgroup analysis, the prognostic significance of serum uPA was highest in patients with HER2-positive disease, followed by the triple-negative subtype; in patients with hormone receptor positive HER2-negative disease tumor, the impact of uPA levels on OS and PFS was not significant (Table 3).

**Table 2:** Overall and progression-free survival according to serum uPA levels and circulating tumor cell counts.

	Median PFS Months [95%-CI]		Median OS Months [95%-CI]	
	≥5 CTCs	<5 CTCs	≥5 CTCs	<5 CTCs
uPA ≥2.52 ng/ml	5.3 [1.7–8.9]	4.2 [1.2–7.2]	7.2 [5.1–9.3]	Not reached (mean 14.1)
uPA < 2.52 ng/ml	5.7 [3.8–7.6]	10.8 [9.1–12.5]	17.8 [10.8–24.8]	Not reached (mean 20.8)

**Table 3:** Prognostic relevance of serum uPA according to the tumor subtype.

Tumor subtype	Overall survival		Progression-free survival	
	Mean/Median months [95%-CI]	p-value	Median months [95%-CI]	p-value
HR-pos HER2-neg	Mean: 15.0 [10.7–19.3] vs. 17.2 [15.4–19.1]; Median not reached	0.319	6.8 [0–14.7] vs. 9.1 [6.9–11.4]	0.387
Triple-negative	Mean: 6.2 [4.1–8.2] vs. 15.9 [12.1–19.7]; Median: 6.2 [1.8–10.6] vs. not reached	0.018	5.3 [2.2–8.4] vs. 7.5 [4.4–10.5]	0.097
HER2-positive	Mean: 9.3 [5.9–12.7] vs. 20.5 [18.6–22.4]; Median: 6.7 [2.5–10.9] vs. not reached	<0.001	4.3 [1.7–6.9] vs. 10.2 [7.3–13.1]	0.001

Abbreviations: HR – hormone receptor, CI – confidence interval.

### Multivariate survival analysis

The variables for the multivariate Cox regression analysis were identified via backward selection. Besides classical prognostic factors, such as grading, line of therapy and number of metastatic sites, biomarkers shown to be associated with survival in the univariate analysis were included in the multivariate model. Elevated uPA, presence of ≥5 CTCs, elevated RAS p21, higher grading and higher line of therapy were independent predictors of shorter OS. Elevated CTC counts, higher line of therapy and negative estrogen status were independent predictors of shorter PFS in the multivariate analysis (Table 4).

**Table 4:** Univariate and multivariate analysis of overall and progression-free survival.

Parameter	Overall survival				Progression-free survival			
	Univariate p-value	Multivariate p-value	HR	95%-CI	Univariate p-value	Multivariate p-value	HR	95%-CI
**uPA**								
Elevated vs. non-elevated	<**0.001**	**0.015**	2.074	1.15–3.74	<**0.001**	0.272	1.276	0.83–1.97
**Menopausal status**								
Pre- vs. Postmenopausal	0.879	—	—	—	0.981	—	—	—
**CTC counts**								
≥5 vs. <5 CTCs/7.5 ml blood	<**0.001**	<**0.001**	3.200	1.70–6.02	**0.001**	<**0.001**	2.193	1.43–3.36
**Therapy line**								
>1^st^ line vs. 1^st^ line	<**0.001**	**0.002**	2.829	1.47–5.45	<**0.001**	<**0.001**	2.964	1.94–4.53
**Grading**								
G3 vs. G1/2	0.183	**0.042**	1.351	1.01–1.81	0.390	0.167	1.151	0.94–1.41
**ER status**								
Positive vs. Negative	0.215	0.189	0.671	0.37–1.22	0.209	**0.020**	0.602	0.39–0.92
**PR status**								
Positive vs. Negative	0.527	—	—	—	0.171	—	—	—
**HER2 status**								
Positive vs. Negative	0.231	0.128	0.634	0.35–1.14	0.973	0.929	0.982	0.66–1.46
**Number of metastatic sites**								
Multiple vs. Single site	**0.010**	0.445	1.321	0.65–2.70	**0.001**	0.117	1.500	0.90–2.49
**Metastatic spread**								
Visceral (+/− bone) vs. bone only	**0.004**	0.429	1.878	0.39–8.97	**0.006**	0.754	0.888	0.42–1.87
**Serum HER2**								
Elevated vs. non-elevated	**0.001**	0.407	1.314	0.69–2.51	0.077	0.083	0.670	0.43–1.05
**Serum RAS p2**								
**1** ≥ 452 pg/ml vs. <452 pg/ml	**0.002**	**0.033**	2.181	1.06–4.48	**0.009**	0.145	1.516	0.87–2.65
**Serum VEGF**								
≥367 pg/ml vs. <367 pg/ml	<**0.001**	0.390	1.284	0.73–2.27	<**0.001**	0.057	1.527	0.99–2.36
**Serum EGFR**								
≥73 ng/ml vs. <73 ng/ml	**0.007**	0.072	0.497	0.23–1.07	0.270	0.422	0.838	0.54–1.29
**Serum TIMP1**								
Elevated vs. non-elevated	<**0.001**	0.419	1.270	0.71–2.27	<**0.001**	0.500	1.167	0.75–1.83
**Serum CAIX**								
Elevated vs. non-elevated	<**0.001**	0.214	1.433	0.81–2.53	<**0.001**	0.149	1.358	0.90–2.06

## Discussion

To our knowledge, this is the largest published analysis of serum uPA levels in metastatic BC. Since the introduction of ELISA-based uPA detection in tumor tissue, a large body of evidence has been obtained that firmly established elevated tissue uPA as an independent prognostic factor in BC^17^. The American Society of Clinical Oncology and the European Society for Medical Oncology incorporated uPA and PAI-1 into their guidelines on early BC^5,6^. Despite that, the commercially available test is not extensively used, mostly due to the requirement for a substantial amount of fresh-frozen tissue and the introduction of well-validated gene signatures that allow the use of formalin-fixed paraffin-embedded samples and are therefore better suitable for routine clinical settings^7,8^. Recently, attempts have been made to develop a reliable assay for uPA detection in fixed tissues^18–20^. Lang *et al*. compared immunohistochemistry (IHC) to measure uPA and PAI-1 with the established ELISA and reported a high correlation between both assays^21^. However, the European Group on Tumor Markers currently discourages the use of IHC- or PCR-based uPA detection for clinical purposes^22^ so as long as the uPA analysis in fixed tissues has not been clinically validated for survival prediction, the biomarker remains a rarely chosen tool to identify high-risk non-metastatic patients in need of chemotherapy^17^.

Beyond detection in the tissue, uPA can be shed by tumor cells, stromal cells or fibroblasts to the blood stream and measured in plasma or serum. In the present study, we aimed at evaluating uPA levels in serum of metastatic BC patients. As with most new biomarkers, an established cut-off value for serum uPA does not exist. A common approach to determine a cut-point is using the sample median, thus dividing the patients into two equally sized groups. For instance, clinical trials on the suitability of serum/plasma VEGF to predict response to anti-angiogenic therapy reported outcomes for patients with above median and below median VEGF levels^23–26^. However, the median value does not always stratify best between patients with good and poor prognosis. In our study cohort, patients with uPA levels above median and below median had similar clinical outcomes (p > 0.1 for OS and PFS). When 1.924 ng/ml, i.e. the upper range of the control group (118 healthy males and females) described in the Manual of the detection kit, was used as cut-off, no significant correlation of uPA levels and survival could be observed either (p = 0.065 for OS and p = 0.206 for PFS). Therefore, we decided to determine the cut-off based on the distribution of uPA and the clinical outcome and performed a ROC analysis for 1-year survival. The optimal cut-off value of 2.52 ng/ml was then chosen using the Youden index; this value is very similar to the upper quartile (2.54 ng/ml).

Interestingly, elevated serum uPA correlated with extent of the disease (p = 0.036 for visceral metastasis and p = 0.016 for multiple metastatic sites) but not with other classical clinical-pathological parameters, like grading or receptor status. With regard to other blood-based biomarkers, significant correlations with serum uPA levels were observed. Patients with high uPA were more likely to present with ≥5 CTCs per 7.5 ml blood (33% vs. 18%, p = 0.008). Whether CTCs shed uPA in substantial amount, remains unclear. The only other study investigating CTCs and serum uPA was conducted in non-metastatic BC^27^. Blood samples from 116 previously untreated patients were collected before surgery. CTC positivity correlated significantly with elevated uPA levels in plasma but not with uPA expression in the tumor tissue, suggesting that CTCs themselves contribute to uPA detected in the blood. Indeed, the authors reported a significantly higher expression of uPA genes in samples with epithelial CTCs compared to CTC-negative samples. Interestingly, CTCs undergoing epithelial-mesenchymal transition (EMT) with higher expression of EMT transcripts than epithelial markers, did not show elevated uPA gene expression. This is surprising, since *in vitro* data showed that EMT induction by uPA receptor (uPAR) requires that cancer cells express not only uPAR but uPA as well^28^. Since the CellSearch system used in the present study is based on an enrichment step using the anti-EpCAM antibody, we cannot exclude the possibility that EMT-CTCs eluded detection by downregulation of epithelial markers^29,30^. Further, elevated serum uPA were associated with high levels of serum VEGF, HER2, RAS p21, CAIX and TIMP1. This is in accordance with previous studies investigating various components participating in the complex crosstalk between signaling pathways. The uPA system is assumed to modulate (neo)angiogenesis by increasing vascular permeability, activating pericellular proteolysis and by enhancing proliferation and migration of endothelial cells^31^. Konukoglu *et al*. examined 66 blood samples from BC patients and found a high correlation between serum VEGF and serum uPA levels^32^. The association between uPA and HER2 is a complex one; while some studies suggested that HER2 specifically promotes the invasive capacity of cancer cells by up-regulating uPA secretion, others found no relationship between levels of both proteins in the blood^33–35^. With respect to the RAS family, uPA and uPAR have been shown to activate the Ras/extracellular signal-regulated kinase (ERK) signaling pathway and to enhance invasive potential of RAS-mutated tumors^36,37^.

Numerous prognostic and predictive factors measured in the tumor tissue have been evaluated for their use also as a ‘liquid biopsy’^38–40^. While soluble forms of such proteins as HER2, VEGF and CAIX can be detected in peripheral blood and elevated levels have been shown to influence prognosis, the currently most widely examined biomarker in metastatic BC are CTCs^41,42^. We have previously shown that serum proteins HER2, VEGF and EGFR predict survival in univariate analysis but lose their prognostic significance in multivariate analysis when CTC status is taken into account as well^38,43,44^. In contrast, we found that elevated levels of serum uPA predicted shorter overall survival both in univariate (p < 0.001) and multivariate (p = 0.015) analysis, showing for the first time that circulating uPA is an independent prognostic factor in metastatic BC. To date, no other trial on the prognostic relevance of serum uPA in metastatic BC has been fully published. Results from three studies are available as conference abstracts only^45–47^. Kang *et al*. evaluated the prognostic and predictive effects of various serum biomarkers, among them uPA, in HER2-positive patients enrolled in the MA.31 trial (NCT00667251) treated by chemotherapy with lapatinib or trastuzumab^45^. Pretreatment serum samples were available for 472 patients; however, it is not clear in how many patients uPA was measured. The authors used the median and the upper limit of norm (1940 pg/ml) as cut-off values. In the abstract presented at the San Antonio Breast Cancer Symposium in 2016, only serum activin A, CAIX, HER2 and TIMP1 were described as prognostic for survival. No statistical details concerning uPA were presented in the abstract. In a smaller study presented at the ECCO European Cancer Conference in 2001, Clarke *et al*. examined pretreatment sera from 244 patients enrolled in a randomized second-line hormone therapy trial^47^. The value of 1.79 ng/ml was used as cut-off for uPA. Time to progression and overall survival were significantly shorter in patients with elevated serum uPA levels than in those with normal levels. Multivariate analysis was not reported in the abstract. In the third, smallest study serum uPA was analyzed in 111 patients with metastatic BC; uPA levels were not prognostically significant^46^. Again, the results have not been fully published since they were reported at the ECCO European Cancer Conference in 2003. Interestingly, in the present trial we could show that the prognostic relevance of uPA differs according to the tumor subtype. Elevated serum uPA predicted poor clinical outcome best in patients with HER2-positive disease and – with regard to overall survival – in triple negative tumors while no statistical significance was observed in the largest subgroup of patients with hormone receptor positive HER2-negative BC (Table 3). This discrepancy seems surprising at first, since evaluation of uPA/PAI-1 in the tumor tissue has been best established in patients with hormone receptor positive HER2-negative disease and the current ASCO clinical practice guideline discourages from its use in HER2-positive and triple-negative disease^5^. A recent analysis including 858 node-negative BC patients as well as several older studies reported that the high prognostic significance of tissue uPA/PAI-1 was independent of the HER2 status^48–50^. Since our study is the first fully published trial on serum uPA in metastatic BC, we can only speculate that elevated uPA levels might contribute to drug resistance to HER2-targeted therapies. Indeed, recent evidence suggests that at least some HER2-positive cancer cells concomitantly express the uPA receptor and that these cells might show stem cell features leading to enhanced invasivess and motility^51^.

In entities other than BC, the association of serum uPA levels and prognosis was investigated in several studies. Miyake *et al*. examined sera from 80 prostate cancer patients and reported that elevated uPA independently predicted reduced OS^52^. In pancreatic cancer, two trials involving 149 and 40 patients have shown that serum uPA levels were significantly associated with clinical outcome^53,54^. In head and neck carcinoma and gastric cancer, uPA levels measured in blood were associated with advanced disease stages but follow-up data are still lacking^55,56^. Beyond prognostication, other possible uses of circulating uPA, such as therapy monitoring or prediction of treatment response, have so far scarcely been explored. In a small Bosnian study, serum uPA was measured during postoperative chemotherapy in 17 patients with ovarian cancer FIGO stage II and III^57^. A significant decrease in uPA levels was observed during therapy; however, survival data were not presented.

## Conclusions

In this prospective translational study, we showed a high prognostic relevance of serum uPA levels in metastatic BC. Most importantly, the impact on survival was independent from other factors, among them the gold standard for blood-based biomarkers, CTCs. Currently, the most exciting potential of soluble markers is the prediction of response to targeted therapy. In case of uPA, it could be hypothesized that patients with elevated serum levels respond to treatment with uPA inhibitors, such as upamostat. Since the clinical trials on uPA inhibitors provided disappointing results, future research should aim at clarifying, whether uPA detection in the blood may identify patients likely to derive a clinical benefit from a targeted strategy.

## Methods

As described in our previous work^38,44,58^, 252 patients with metastatic BC from nine German Breast Cancer Centres were enrolled in this prospective, multicentre, open-label, non-randomized study. All patients were at least 18 years old, provided an informed consent and were scheduled to receive first or later line of systemic therapy for metastatic disease. Treatment was chosen according to institutional and national standards and was independent of the participation in the present study. Blood samples were obtained before start of treatment. Patients with a co-existing or previous second malignancy (except *in situ* carcinoma of the cervix or adequately treated cutaneous basal cell carcinoma) could not participate in the study. Response to therapy was evaluated according to institutional guidelines usually by computed tomography every 12 weeks.

### Quantitative analysis of serum uPA

uPA was quantified by a commercially available sandwich ELISA immunoassay (Item No. 06489892, Oncogene Science, formerly Siemens Medical Solutions Diagnostics, now Wilex Inc., MA, USA) used to quantify pro-, high molecular weight and low molecular weight uPA found in human serum or plasma. This method uses two monoclonal antibodies to human uPA immobilized on the interior surface of microtiter plate wells as the capture reagents. First, the specimen is placed and incubated in the coated well. This allows the antigen to bind to and be immobilized by the Capture Antibody. Afterwards, the antigen is reacted with the rabbit antiserum used as uPA detector, which is then bound with a goat-anti-rabbit IgG-horseradish peroxidase Conjugate enabling the exact measurement of the amount of antigen-bound Detector Antibody. The incubation with o-phenylenediamine substrate leads to color development that is quantified using spectrophotometry and allows the quantitation of captured uPA. The uPA concentration is estimated from the standard curve. Samples were analyzed in duplicate. According to manufacturer’s instructions, the mean recovery of uPA using the assay in 118 healthy controls of both sexes was 1.192 ng/ml serum (range: 0.459–1.924 ng/ml).

### Detection of other biomarkers

CTCs were detected using the CellSearch™ system (Veridex LLC, NJ, USA) as described previously^38,43,44,58^. Briefly, 7.5 ml peripheral blood were collected into CellSave Tubes and processed according to manufacturer’s instructions. The detection assay consists of an immunomagnetic enrichment step employing immunomagnetic beads coated with anti-epithelial cell adhesion molecule (EpCAM) antibody, followed by staining with several antibodies. A CTC is defined as a CD45-negative cytokeratin-positive cell with a DAPI-stained nucleus. In the current study, CTC-positive patients were defined as those with at least five tumor cells per 7.5 ml blood. Serum HER2 was determined using a commercially available ELISA (Martell Diagnostic Laboratories, Roseville, MN, USA; formerly Wilex Inc, Cambridge, MA, USA), as described previously^38^. This assay measures the extracellular domain of the HER2 protein and has been cleared by the Food & Drug Administration (FDA) with the recommended cut-off of 15 ng/ml. Serum EGFR^43^, VEGF^44^, RAS p21^58^, TIMP1^40^ and CAIX^40^ were quantified by ELISA as described in our previous works.

### Statistical analysis

Chi-squared test was used to examine the relationship between uPA levels and other parameters, such as clinical and histological factors as well as other blood-based biomarkers. Survival intervals were measured from the time of blood sampling to the time of death or of tumor progression, defined as the first radiological, histological and/or clinical diagnosis of progression. Receiver operating characteristic (ROC) analysis was performed to assess possible cut-off values. The optimal cut-off has been determined using the Youden index. The log-rank test was used to evaluate the univariate significance of the clinical, histological and blood-based parameters. For multivariate analysis, Cox regression analysis was performed. The reported p-values are two-sided; values ≤ 0.05 were considered significant. Statistical analysis was performed by the statistical package R version 3.5.1 (R Core Team (2018). R: A language and environment for statistical computing. R Foundation for Statistical Computing, Vienna, Austria) and SPSS (SPSS Inc., Chicago, IL, USA). The study was performed and the manuscript prepared according to the REporting recommendations for tumor MARKer prognostic studies (REMARK) criteria on reporting of biomarkers^59^. The prognostic relevance of serum uPA was the primary question of the present study.

### Ethical approval

All procedures performed in this study were in accordance with the ethical standard of the institutional and national research committee and with the 1964 Helsinki declaration and its later amendments or comparable ethical standards. The study was approved by the local ethical committees of participation institutions: University of Hamburg, University of Duesseldorf, University of Tuebingen, University of Erlangen, University of Duisburg-Essen, University of Freiburg, University of Heidelberg, University of Munich and University of Regensburg.

### Informed consent

Informed consent was obtained from all individual participants included in this study.

## Data Availability

The datasets generated during the current study are available from the corresponding author on reasonable request.
